# Endovascular treatment of pulmonary artery aneurysm: Single case experience

**DOI:** 10.1016/j.radcr.2023.09.021

**Published:** 2023-10-07

**Authors:** Adriana Ilardi, Mariangela Clemenza, Sebastiano Piana, Diego Meo, Viviana Lentini, Vincenzo Magnano San Lio, Giuseppe Giordano

**Affiliations:** a Radiology Unit 1, AOU Policlinico “G. Rodolico - San Marco”, PO “G. Rodolico”, University of Catania, Catania, Italy; b Diagnostic and interventional Radiology Unit ARNAS Garibaldi - Nesima - Catania, Italy

**Keywords:** Peripheral pulmonary artery aneurysm, Coil embolization, CT angiography, Chest

## Abstract

A peripheral pulmonary artery aneurysm (PAA) is a dilatation involving all 3-vessel wall layers (the intima, media, and adventitia) of a distal pulmonary artery. It represents a rare but potentially life-threatening condition. There are only some reviews of transcatheter embolization of unruptured idiopathic peripheral PAAs. Association with cardiac diseases, infections, vascular anomalies, pulmonary hypertension, and vasculitis has been noted. We report a case of a 38-year-old woman, with a history of third-degree atrioventricular (AV) block, treated with pacemaker placement, who presented a PAA in the left pulmonary lobe. Transcatheter coil embolization was performed, using a triple coaxial catheter system (a 6F outer, a 5F intermediate, and a 2.4F inner catheter) to prevent rupture and the aneurysm was successfully embolized. Although there is no consensus on the treatment for unruptured idiopathic peripheral PAAs, transcatheter embolization may be a promising treatment option.

## Introduction

Pulmonary artery aneurysms (PAAs) are uncommon. An incidence rate of 0.007% was reported in autopsies. A peripheral pulmonary artery aneurysm (PAA) is a dilatation involving all 3-vessel wall layers (the intima, media, and adventitia) of a distal pulmonary artery [1]. PAAs are categorized as congenital or acquired. Idiopathic aneurysms are a subgroup of acquired aneurysms that arise in patients without risk factors of PAAs (trauma, connective tissue disorders, vasculitis, or pulmonary hypertension) [2]. We describe the finding of a PAA in a 38-year-old woman with a history of pulmonary embolism. Since the absence of guidelines for managing the case mentioned above, we aim to define a possible approach to a potentially life-threatening rare event.

## Case report

**Fig. 1:**
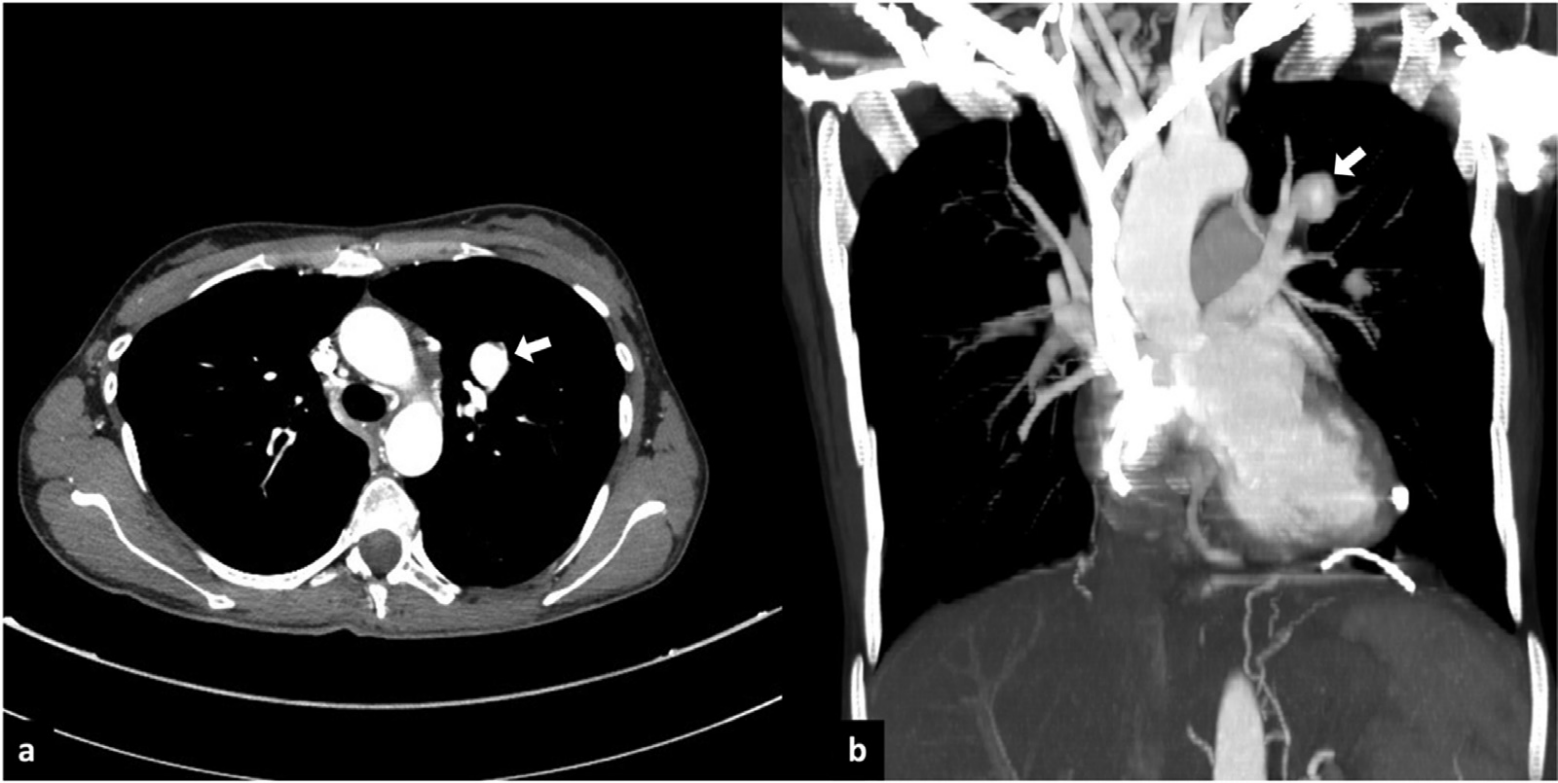
Computed tomography angiography (CTA) images. (A) Axial and (B) coronal maximum intensity projection (MIP) reconstruction views show the presence of an aneurysmal sac originating from a segmental branch of the left pulmonary artery (white arrows).

**Fig. 2:**
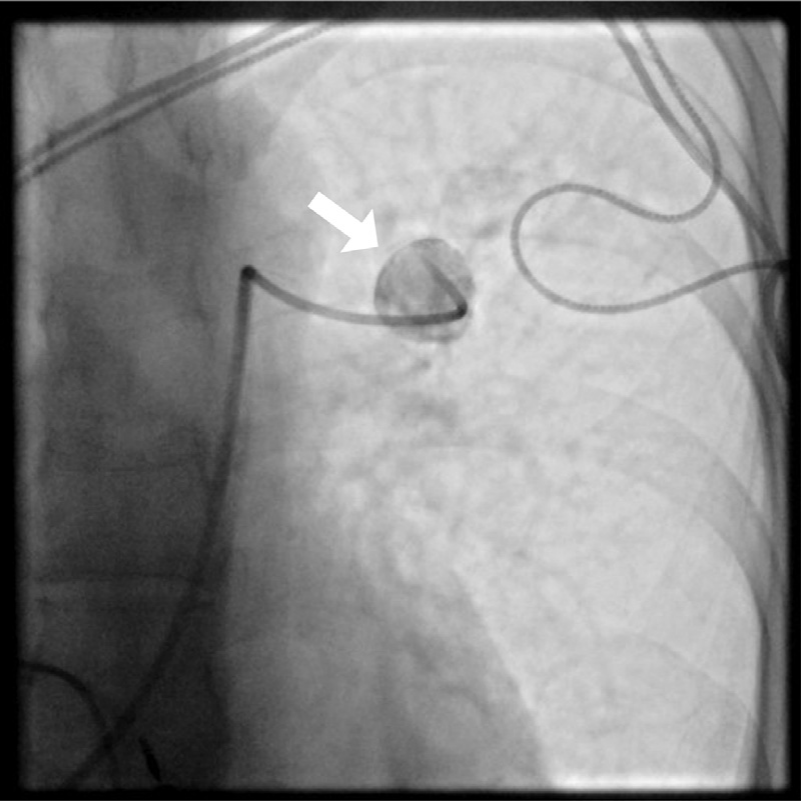
Selective angiogram performed with a 5Fr catheter shows the aneurysmal sac (arrow).

**Fig. 3:**
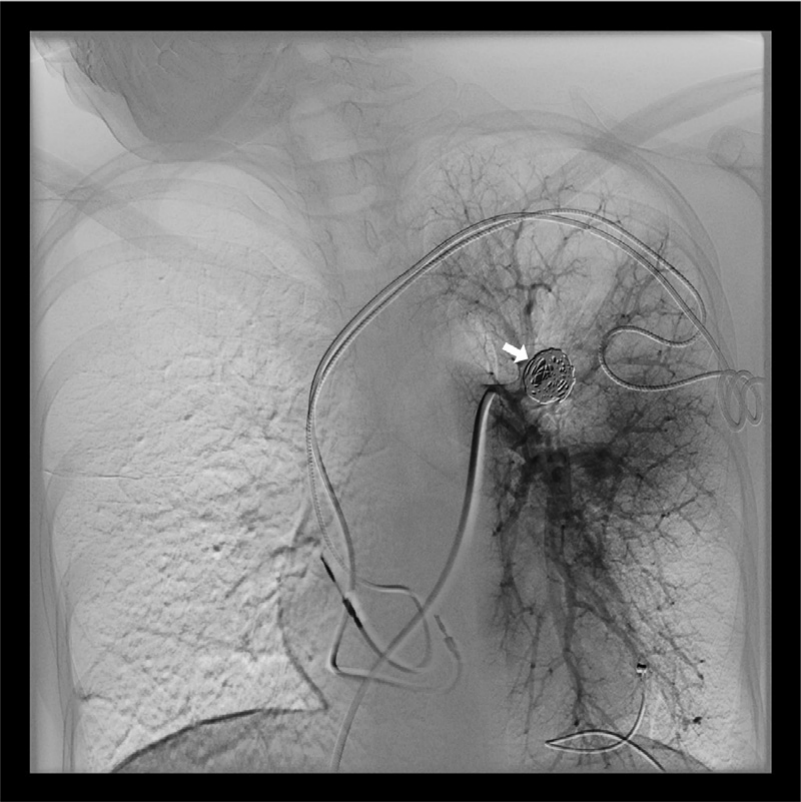
Selective angiogram of the left pulmonary artery following embolization. No inflow into the aneurysm was detected (arrow). The patency of the remaining branches of the left pulmonary artery was regular (arrowheads).

A 38-year-old woman presented a medical history of third-degree atrioventricular (AV) block, treated with 2 pacemaker placement, and previous episodes of pulmonary embolism. When she was affected by COVID-19 infection, she was admitted to the thoracic surgery department, due to her increased thrombotic risk, although she did not present any COVID-19 pneumonia. She did not have any CT scans during her initial presentation from COVID-19. A chest CT scan, 2 months after the infection, showed a round lesion in the left upper lobe. This finding was consistent with a PAA (Fig. 1). The lesion was treated using the triple coaxial catheter technique, under local anesthesia and intravenous sedation. This technique consisted of a 6F 65 cm guiding catheter as the outer catheter and a 5F 100 cm angiographic catheter (with shape Berenstein) as the middle catheter and a 2.4 F 130 cm microcatheter as the inner catheter to help navigation. The 2.4 F microcatheter was advanced selectively into the aneurysmal sac. Two Penumbra Ruby Coil (Penumbra Inc., Alameda, California), 16 mm × 60 cm and 14 mm × 60 cm, were put inside the sack of the aneurysm to perform transcatheter embolization (Fig. 2). A following angiography demonstrated complete exclusion of the aneurysm with no significant loss of vascular supply (Fig. 3). The patient did not show any periprocedural complications.

## Discussion

Pulmonary artery aneurysms represent a potentially life-threatening condition, although their low incidence, and could present a challenge for timely diagnosis and treatment. A dilatation of the distal pulmonary artery involving all 3-vessel wall layers (intima, media, and adventitia) is defined as PPA. The most accepted cutoff to describe a PAA is a diameter 1.5 times bigger than the greatest diameter of a proximal segment of the same artery. Although the real occurrence of peripheral PAA is uncertain, autopsy research proved an incidence of 0.007% [3].

An anatomic classification differentiates peripheral PAA (11%), arising from the distal vessels (lobar, segmental, and subsegmental branches), and central PAA, originating from a major pulmonary vessel (89%) [4]. The branches of the left pulmonary artery represent the most common sites of peripheral PAA, as in our patient.

Another morphological classification distinguishes saccular, which is defined as a noncircumferential outpouching of the vessel wall, and fusiform aneurysms, which include the entire circumference of the vessel [2].

Symptoms are nonspecific and may overlap with different conditions. In addition, patients may remain asymptomatic. Reported symptoms include hemoptysis, which might be suggestive of imminent aneurysm rupture, shortness of breath, chest pain, palpitations, or syncopal episodes [5]. The pathophysiology is based on LaPlace's law, which states that the aneurysmal diameter increases with increased arterial wall tension.

Etiologies of PAA can be distinguished as congenital or acquired. A subgroup of acquired etiologies is represented by idiopathic aneurysms. Congenital causes were recognized as the main reason for PAA formation. More than 50% of all cases have been associated with congenital heart disease (in decreasing order, persistent ductus arteriosus, ventricular septal defects, and atrial septal defects). Other common congenital causes of PAA include diseases affecting connective tissue and vessels (Ehlers-Danlos syndrome, Marfan syndrome, and cystic medial necrosis) [6]. Acquired etiologies comprise trauma and iatrogenic injury (Swan-Ganz catheter placement, cardiothoracic surgery, chest tube placement, catheter-directed angiography, lung biopsies, and percutaneous ablation, infection) [7]. Pulmonary artery hypertension (PAH) is considered another relatively common cause of PAA. Another risk factor for aneurysm formation is chronic pulmonary embolism, which could act through direct pulmonary arterial wall injury or poststenotic arterial dilatation. Recurrent pulmonary embolism was a condition present in our patient. Another rarer cause of PAA is vasculitis. Among these, the most frequently reported was Behcet's disease (a chronic multisystem vasculitis hallmarked by oral and genital ulcers and uveitis).

PAAs should be distinguished from pulmonary arteriovenous malformations (AVM), due to the risk of paradoxical embolism of AVM. AVM are abnormal direct connections between the pulmonary arterial and venous circulation that bypasses the capillaries developing a high-flow system. This lesion could mimic an aneurysmal sac of PAA at imaging [8]. A key diagnostic finding of AVM in angiography is the presence of draining veins in AVM.

The mortality rate associated with rupture was about 50%-100%; death is mainly related to aspiration and asphyxia following intrapulmonary hemorrhage [6]. PAA also could result in the dissection of the pulmonary artery and sudden cardiac death. Therefore, early diagnosis and treatment are significant for patient survival and optimal outcomes.

PAA may be suspected at standard radiography when a hilar enlargement, lung nodule, or pulmonary mass is found. Computed tomography angiography (CTA) is the mainstay of imaging for diagnosis and follow-up of PAA, assessing the presence, size, location, and characteristics of PAAs and providing information on the etiology of aneurysms.

Catheter-directed angiography represents the gold standard for the diagnosis of PAA. This technique describes the extension of vascular involvement, assesses the right-sided cardiac pressures, and allows simultaneous endovascular treatments. Major limitations are not to provide information regarding extra-luminal structures, important to determine etiology, and to be an invasive exam [2].

Once a PAA is diagnosed, the next step is to establish the appropriate treatment. There are no clear guidelines for the best management.

Conservative treatment appears reasonable for asymptomatic patients with stability in PAA diameter and without significant PAH [4].

In patients with PAH, surgical treatment (aneurysmorrhaphy, lobectomy, bilobectomy, aneurysmectomy, and pneumonectomy) should be considered. Although specific guidelines for endovascular versus surgical treatment of PAA do not exist, many case reports support first-line endovascular treatment with coil embolization (or vascular plugs), when feasible, due to the high morbidity and mortality rates of surgical treatment. Nevertheless, central fusiform aneurysms require surgical management [9]. Intrasaccular embolization with coils preserves pulmonary arteries distal to a PAA while sparing pulmonary function. Embolization performed proximal and distal to the PAA neck represents a valid alternative in case of unfeasible or incomplete intrasaccular embolization or increased risk of rupture. The risks of embolization include nontarget embolization, arterial dissection and thrombosis, end-organ ischemia, and contrast-induced nephropathy. In ruptured PAA surgery is mandatory as the only possible life-saving treatment. Dissection, in case of reasonable preoperative morbidity, represents another indication for surgery.

There is no sufficient evidence about the rate of rupture of idiopathic peripheral PAAs, although high mortality rates have been described for solitary peripheral PAAs [9]. Therefore, a treatment to prevent rupture should be considered.

In the present case, intrasaccular embolization was achieved using coils. The patient had not any complications.

## Conclusions

Despite the infrequency of peripheral PAAs, prompt recognition and diagnosis are imperative for both radiologists and clinicians, due to the high mortality rates related to rupture. Accurate guidelines for the management of PAAs are still needed, to help clinicians in defining when intervention is appropriate. Endovascular coil embolization is the cornerstone of treatment due to its minimal invasiveness and lower rates of complications compared to surgery, although further studies are required.

## Patient consent

This is to state that the patient gives her full permission for the publication, reproduction, broadcast, and other use of photographs, recordings, and other audio-visual material of herself (including of her face) and textual material (case histories) in all editions of the above-named product and in any other publication (including books, journals, CD-ROMs, online, and internet), as well as in any advertising or promotional material for such product or publications.

She declares, in consequence of granting this permission, that she has no claim on ground of breach of confidence or any other ground in any legal system against authors and their agents, publishers, successors and assigns in respect of such use of the photograph(s) and textual material (case histories).
